# Electroacupuncture pretreatment alleviates myocardial injury through regulating mitochondrial function

**DOI:** 10.1186/s40001-020-00431-4

**Published:** 2020-08-01

**Authors:** Chunai Wang, Xi Liang, Yan Yu, Yulan Li, Xiaohui Wen, Min Liu

**Affiliations:** 1 Gansu Provincial Hospital of Traditional Chinese Medicine, No. 424, Guazhou Road, Qilihe District, Lanzhou, 730050 Gansu China; 2 The First Hospital of Lanzhou University, Lanzhou, Gansu China; 3 Gansu University of Chinese Medicine, Lanzhou, Gansu China; 4 Affiliated Hospital of Gansu University of Chinese Medicine, Lanzhou, Gansu China

**Keywords:** Bupivacaine, Electroacupuncture preconditioning, Cardiac mitochondria, Neiguan point, Lipid emulsion

## Abstract

**Background:**

Electroacupuncture is well known for its advantageous neuroanalgesic and therapeutic effects on myocardial ischemia–reperfusion injury. The purpose of the present research was to verify whether electroacupuncture can alleviate bupivacaine-induced myocardial injury.

**Methods:**

Specific pathogen-free Wistar rats were used to establish the bupivacaine-induced myocardial injury model. Western blot, PCR, transmission electron microscope and enzyme-linked immunosorbent (ELISA) methods were used to evaluate bupivacaine-induced structure injury and dysfunction of the mitochondria as well as the alleviating effects of lipid emulsion, acupoint injection, and electroacupuncture pre-treatment of the oxidase stress response.

**Results:**

Bupivacaine caused structural damage, degradation, and swelling of mitochondria. Furthermore, it reduced adenosine triphosphate (ATP) synthesis and impaired energy metabolism in the mitochondria. Structural and functional impairment of the mitochondria was alleviated via lipid emulsion injection, acupoint injection, and electroacupuncture pre-treatment. Electroacupuncture pre-treatment of PC6 yielded a greater alleviating effect than others approaches. Following electroacupuncture pre-treatment of PC6 point, the number of mitochondria increased; apoptosis was reduced, enzymatic activity of cytochrome C oxidase (COX) and superoxide dismutase and expression of uncoupling protein 2, voltage-dependent anion channel 1, and Bcl 2 were upregulated and SLC25A6, MDA levels were downregulated. Additionally, our findings indicated that electroacupuncture pre-treatment of PC6 point exerted an effect on the mitochondria via the mitochondrial-transcription-factor-A/nuclear-respiratory-factor-1/proliferator-activated-receptor-gamma-coactivator-1 pathway.

**Conclusion:**

The present study revealed that electroacupuncture pre-treatment of PC6 could effectively alleviate bupivacaine-induced myocardial mitochondrial damage, thereby providing a theoretical basis for clinical studies and applications of this treatment method.

## Background

Local anesthesia is often performed during surgical procedures to reduce the suffering of patients and improve surgery success rates [1]. However, local anesthesia can also lead to complications, resulting in inevitable adverse effects. Local anesthesia affects the central nervous system and the cardiovascular system and may therefore lead to neurotoxicity [2], resulting in nerve injuries and pathological changes in the peripheral nerves. Deaths from local anesthesia are mainly attributed to adverse effects on the cardiovascular system, which include contractile dysfunction and cardiac arrhythmia [3], and are mainly characterized by hemodynamic changes [4].

Bupivacaine is an amide, which is a long-acting local anesthetic commonly administered in various procedures, such as labor analgesia and total knee arthroplasty. Bupivacaine has the advantage of rapid onset of action and is relatively safe at the recommended dosage [5]. However, overdose or accidental intravasal application of bupivacaine can result in significant adverse effects. For every 1000 peripheral nerve blocks, 0.04 to 1.8 cases of myocardial toxicity are likely to occur, leading to severe symptoms [6]. Bupivacaine reportedly induces muscle toxicity in skeletal muscles [5]. Bupivacaine-induced cardiotoxicity may result in cardiac arrhythmia, poor myocardial contractility and cardiac arrest due to circulatory collapse [7, 8]. Sztark et al. reported that bupivacaine may directly inhibit mitochondrial respiratory chain complex I (MRCC-I), alter mitochondrial membrane structure, increase proton permeability in the mitochondrial inner membranes (MIMs), induce loss of mitochondrial calcium, and reduce mitochondrial membrane potential (MMP) [9], thereby affecting the respiratory function and energy production of the mitochondria and eventually leading to mitochondrial dysfunction.

Evidently, bupivacaine inhibits MRCC-I activity in a dose-dependent manner, accounting for alterations in mitochondrial energy fluxes [10]. Bupivacaine negatively regulates fatty acid oxidation and oxidative phosphorylation and inhibits the activity of mitochondrial carnitine transferases, thereby reducing the synthesis of adenosine triphosphate (ATP) required for myocardial contractility [7]. Bupivacaine also inhibits the aerobic respiration of the mitochondria; specifically, decoupling of oxidative phosphorylation alters MMP and inhibits mitochondrial respiration, resulting in the production of reactive oxygen species (ROS) in the mitochondria and triggering the mitochondrial pathway of apoptosis [11]. Lipid emulsion is widely used to alleviate bupivacaine-induced myocardial toxicity because this technique can enhance mitochondrial metabolism and improve myocardial contractility [12, 13]. Similarly, puerarin isolated from the traditional Chinese medicine radix puerariae has demonstrated a protective effect on myocardial infarction by targeting the mitochondria [14].

Over the past three millennia, acupuncture has exhibited significant potential in clinical studies of various diseases in China and other Asian countries. Electroacupuncture (EA) is a combination of acupuncture and electrical stimulation [15]. Because EA allows for more precise parameter tuning, it has been widely applied in clinical studies [16]. Previous studies have shown that EA can regulate mitochondria autophagy, inhibit NLRP3 inflammasome activation, and alleviate myocardial injury [17, 18]. EA can also serve as an adjuvant therapy for various types of pain with fewer side effects; glial cells may contribute to these analgesic effects [19]. Besides, EA can also affect the secretion of proinflammatory cytokines and vascular endothelial growth factor by regulating endocrine hormones [20].

Acupuncture-assisted anesthesia, such as ST36 stimulation is commonly used in clinical studies to reduce the required dose of bupivacaine and thus avoid its side effects [21]. Similarly, acupuncture of ST40 can exert a protective effect on heart structure and function by regulating the metabolism of lipid emulsions [22]. The Neiguan point (Pericardium-6, PC6) is located on the medial surface of the forelimb between the tibia and the ulna. Researchers have verified that PC6 plays a vital role in cardiovascular diseases when stimulated. Reportedly, the protective effects of PC6 EA are predominantly achieved via the regulation of various signals of mitochondrial energy metabolism [23]. In present study, we explored the effects of PC6 stimulation on structure and function of mitochondria.

## Materials and methods

### Materials

The primary materials used in the present study were as follows: fetal bovine serum (FBS), bupivacaine, lipid emulsions, puerarin injections, pentobarbital sodium, the RNeasy Mini Kit (QIAGEN), the QuantiNova SYBR Green PCR Kit (QIAGEN), the QuantiNova Reverse Transcription Kit (QIAGEN), Anti-UCP2 (GeneTex), Anti-NRF1 (GeneTex), Anti-SLC25A6 (GeneTex), Anti-mtTFA (GeneTex), Anti-VDAC1, Anti-PGC-1 (Abcam), Anti-Bcl-2 (Abcam) and ATP assay kit (Abcam, ab83355). JC-1 kit (Beyotime, C2006), Fluo-3 AM (Abcam), DCFH-DA (Solarbio), Rat COX ELISA kit (CUSABIO), MDA ELISA Kit (Elabscience).

### General animal and animal group treatment conditions

Animal experimentation was carried out in the present study in accordance with the requirements of Committee of the Animal Protection and Utilization Institute. This study was approved by animal experiment ethics of Gansu University of Traditional Chinese Medicine (No. 2018-018) and complied with the Declaration of Helsinki. Male specific pathogen-free Wistar rats (8 weeks old, weighing 300 ± 10 g) were provided by Shanghai Experimental Animal Co., Ltd. (Shanghai, China). Each of these animals was anesthetized and placed in the supine position on a surgical platform, and three needle electrodes were placed under the skin to produce a 12-lead electrocardiogram (ECG) and monitor other basic conditions, such as blood pressure and heart rate.

The experimental rats were divided into six groups as follows: control group (*n* = 8) rats intravenously infused with 3 ml of 0.9% physiological saline solution (kg/min) and killed after 30 min; bupivacaine group (*n* = 16) rats intravenously infused with physiological saline solution for 30 min and then infused with 0.5% bupivacaine to induce cardiac arrhythmia or death (standard criteria for cardiac arrhythmia); lipid emulsion group (*n* = 16) rats subject to continuous intravenous infusion with 3 ml of 20% lipid emulsion for 30 min, followed by infusion with bupivacaine to induce cardiac arrhythmia or death; puerarin group (*n* = 16) rats infused with bupivacaine after being injected with 0.1 ml of puerarin at PC6 on both forelimbs; EA-PC6 group and EA-ST36 group (*n* = 16) rats infused with bupivacaine after 30 min of EA stimulation (longitudinal wave: 2/10 Hz; current intensity: 2 mA; pulse width: 0.2 ms) at PC6 and ST36, respectively.

The rate and duration of infusion and lipid emulsion administration were determined according to a study reported by Weinberg [24]. Cardiac arrhythmia was assessed by an electrocardiographer on the basis of premature ventricular contractions (PVC) or ventricular tachycardia (VT) with the duration of the QRS complex prolonged. At the conclusion of the experiment, the rats were euthanized and subjected to retrograde perfusion followed by immediate resection of their hearts for subsequent analyses.

### Isolation of mitochondria

Myocardial mitochondria were isolated using a previously published method with some modifications [25]. The rats were euthanized with pentobarbital sodium, and their hearts were harvested, excised into small pieces, and homogenized. This process was followed by the removal of cell debris. Then, the supernatant was centrifuged at 13,000*g* for 10 min to isolate the mitochondria.

### Mitochondrial membrane potentials (MMP) assay

MMP was measured with a JC-1 kit (Beyotime, C2006) according to the manufacturers’ instructions. The 1× JC-1 working solution was added to the purified mitochondria with an appropriate proportion and the results were analyzed using a fluorescence spectrophotometer. The excitation wavelength was 458 nm, and the emission wavelength was 590 nm.

### Determination of calcium ion levels in mitochondria

Mitochondrial calcium ion concentrations were measured using Fluo-3 AM in accordance with previous study [7]. Fluo-3 AM was added to resuspended mitochondria and incubated for 1 h. Then, the Fluo-3 AM was removed. Fluorescence intensity was analyzed by flow cytometry after the mitochondria were subjected to Triton X100, calcium chloride and EDTA, respectively.

### Reactive oxygen species measurement

Cardiomyocytes were isolated from the SD rats, and their ROS levels were evaluated by incubating the cells in DCFH-DA. After 30-min incubation, the cells were subjected to washed, and resuspended. Subsequently, the results were analyzed using a microplate reader at an excitation wavelength of 488 nm and an emission wavelength of 525 nm.

### ATP content

Myocardial ATP levels were measured using a commercial assay kit. Briefly, tissues were lysed and centrifuged at 12,000*g* and 4 °C for 5 min. The resulting supernatant was harvested for subsequent assays. Mitochondrial ATP content was analyzed by colorimetry using phosphomolybdic acid.

### Enzyme-linked immunosorbent assay (ELISA)

After being homogenized in phosphate-buffered saline, the tissue samples were centrifuged to obtain the supernatant for subsequent measurements. All experimental procedures were carried out in accordance with the manufacturers’ instructions provided with the commercial kits. Standard samples and test samples were placed in wells intended for blank, standard, and test samples, respectively. After being incubated at 37 °C for 30 min, each well was washed and incubated with the enzyme-labeling reagent. Then, the wells were washed again and subjected to color development. After the reaction was terminated, the absorbance value of each well was measured to estimate the sample concentration. This experiment was repeated three times.

### qPCR

Cardiac muscle tissues were rapidly harvested and immersed in liquid nitrogen to extract RNA for subsequent analyses. Experimental procedures were carried out in accordance with the instructions provided with the commercial kits. The purity and quality of the resulting RNA samples were determined alongside the removal of DNA, followed by a reverse transcription PCR (RT-PCR) assay. Primer sequences for the target genes are listed in Table 1.

**Table 1:** Primer sequences

Genes	Forward	Reverse
GAPDH	AATGGTGAAGGTCGGTGTGAAC	AGGTCAATGAAGGGGTCGTTG
Bcl-2	ATGATAACCGGAGATCGTG	GACGGTAGCGACGAGAGAAG
VDAC1	CACCAAAGTGAACGGCAGTC	TGCTCCCTCTTGTACCCTGT
UCP2	GCCAACCTCATGACAGACGA	AGGAAGGCATGAACCCCTTG
PGC-1α	CATGTGCAGCCAAGACTCTG	GTGAGGACCGCTAGCAAGTT
NRF1	GCTAATGGCCCAGATATGGAGT	CGTAAGCTCTGCCTGGTTGT
mtTFA	GGAATCAAGACTGTGCGTGC	AGAAACTGCAATGGCTCTGC
ANT1	GCTAACCAACCCACTGTCCT	ATGCCACCGCTAACAAGACAT

### Western blot

After the tissue samples were homogenized, the supernatant was obtained for the purpose of isolating total proteins using a protein extraction kit. After the protein concentration was measured, the total proteins were loaded in equal amounts and separated via sodium dodecyl sulphate polyacrylamide gel electrophoresis. Subsequently, the proteins were transferred onto a polyvinylidene fluoride membrane, which was then incubated with special primary antibodies followed by secondary antibodies for 1 h at 37 °C. Western blot images were obtained via the enhanced chemiluminescence method. Grayscale analysis was performed on target protein bands using Image J software, and the results were analyzed statistically.

### Transmission electron microscopy

Myocardial tissues were sequentially fixed with 2.5% glutaraldehyde and 1% citric acid then dehydrated in an acetone gradient and embedded in resin. Then the embedded tissues were dried rapidly prior to sectioning. Next, tissue sections were stained and imaged under an electron microscope. Mitochondrial injuries were assessed using the Flameng score [26]. Five microscopic fields were randomly selected to obtain the mean Flameng score for each group and the mitochondria were graded according to the following criteria: Grade 0 (score 0), mitochondria with normal ultramicrostructure and intact granules; Grade I (score 1), mitochondria with basically normal ultramicrostructure and partial loss of granules; Grade II (score 2), swollen mitochondria with transparent matrices; Grade III (score 3), mitochondria with transparent matrices and fragmented cristae or formation of flocculent densities in their mitochondrial matrices; and Grade IV (score 4), mitochondria lacking matrix with fragmented cristae and disrupted outer membranes.

### Statistical analysis

The statistical analyses of the present study were performed using SPSS 20.0 software, and the data were expressed as means of the scores yielded by triplicate experiments. Multiple comparisons between groups were carried out using one-way analysis of variance (ANOVA). *P*-values < 0.05 were considered to indicate statistically significant results.

## Results

### Protective effect of different treatments on bupivacaine-induced myocardial injury

**Fig. 1:**
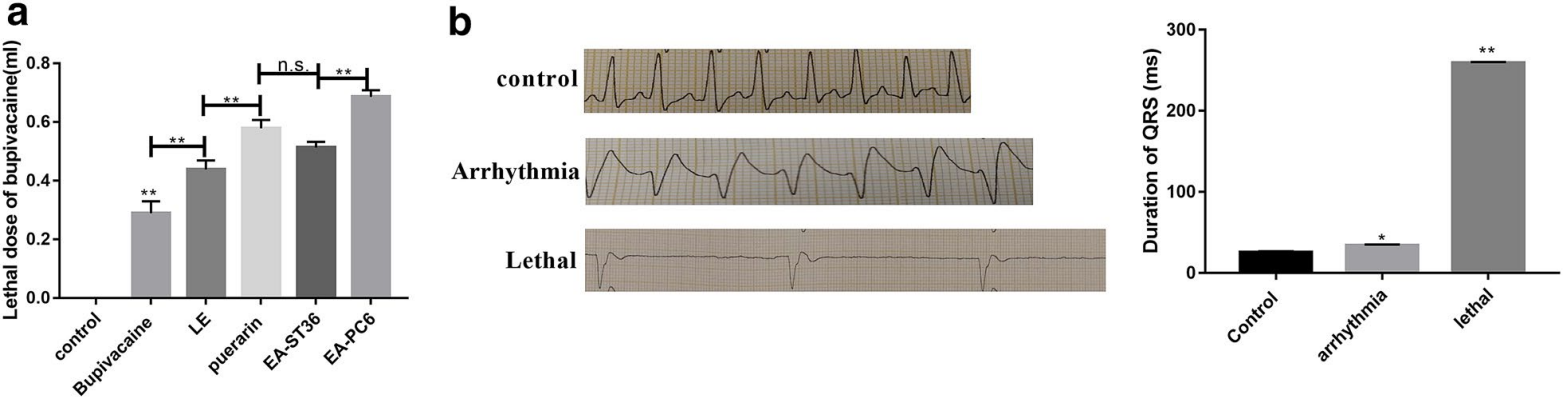
Protective effect of EA pre-treatment on myocardial mitochondria. **a** Lethal doses of bupivacaine in different experimental groups. **b** Changes of QRS duration

Previous studies have demonstrated that EA pre-treatment exerts an alleviating effect on bupivacaine-induced toxicity [8]. As shown in Fig. 1a, significant differences in the lethal dose of bupivacaine were observed between different groups, among which the EA pre-treatment of PC6 corresponded to the highest lethal dose of bupivacaine. The lethal dose of bupivacaine in EA-PC6 was higher than EA-ST36 and puerarin injection at PC6. This finding suggested that EA-PC6 had the greatest alleviating effect against myocardial toxicity. Intravenous injection with lipid emulsions yielded a relatively poor alleviating effect, but still outperformed the bupivacaine group. The results showed that bupivacaine significantly prolonged the duration of the QRS complex. The duration of QRS complex was 26 ms, 32 ms, 260 ms in control, arrhythmia, and lethal groups, respectively. The difference between the control and arrhythmia groups was significant (*P* < 0.05). The difference between the QRS complex durations of the lethal and control groups was extremely significant (*P* < 0.01; Fig. 1b).

### The effect of EA pre-treatment on mitochondrial structure

**Fig. 2:**
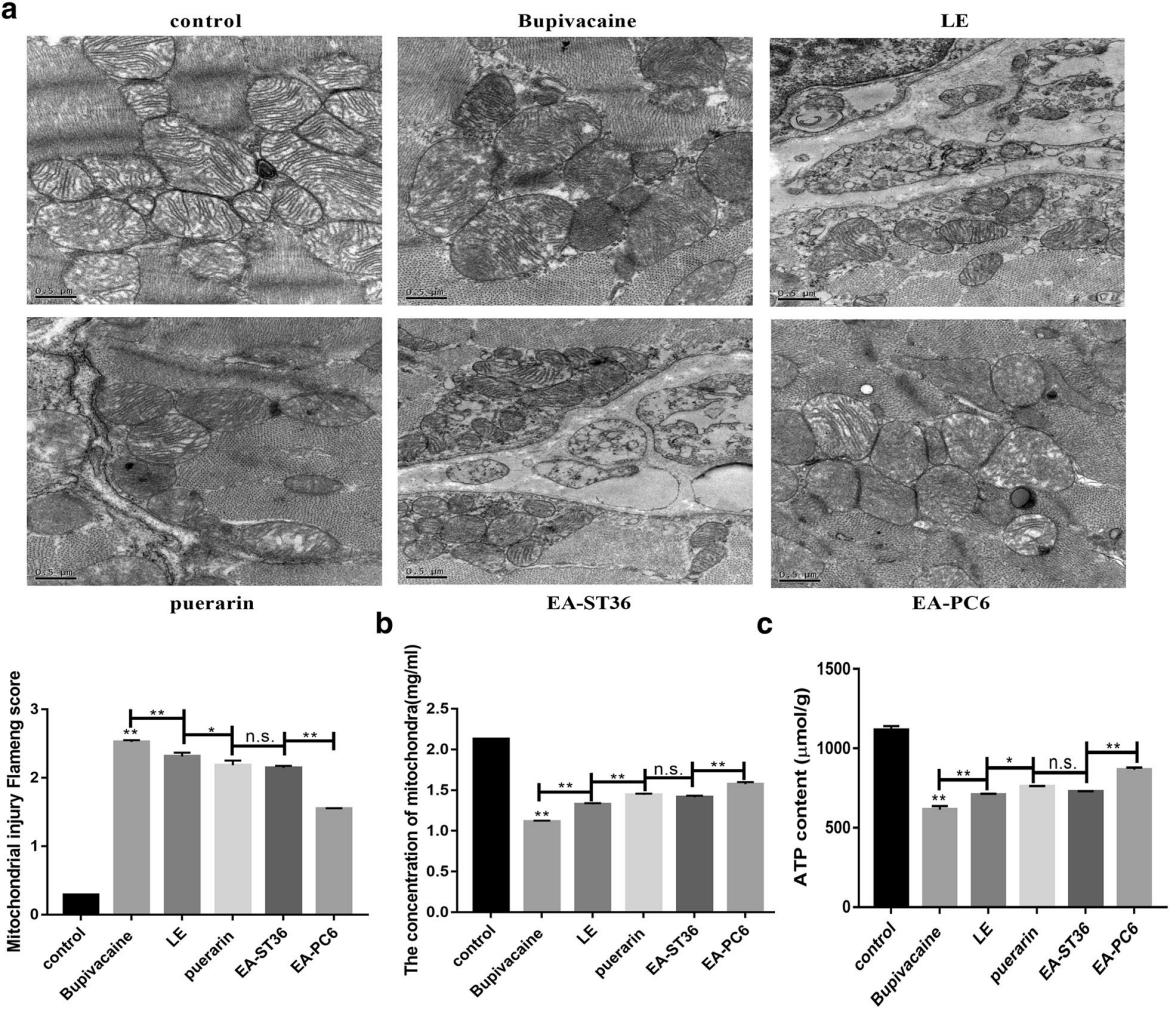
Effects of EA pre-treatment on mitochondrial structure. **a** Ultramicrostructure and damage score of mitochondria. **b** The concentration of mitochondria. **c** The content of ATP

Structural and functional damage to the mitochondria is the main mechanism underlying myocardial injury. The control group rats in the present study exhibited densely packed myocardial fibers with clearly visible sarcomeres and abundant mitochondria. Their mitochondrial structure was intact with a typical oblong shape and continuous cristae, as well as intact membranes and granules (Fig. 2a). The rats in the bupivacaine group displayed mitochondrial degradation with swollen, fragmented, and even missing cristae. The lipid emulsion group, the puerarin group, and the EA pre-treatment group exhibited increasingly alleviated mitochondrial injuries. The Flameng scores showed that bupivacaine was associated with the least mitochondrial damage in the EA group rats. In addition, there were significant differences in the Flameng scores between different groups (*P* < 0.01). And the therapy effect of EA-PC6 was significantly preceded other treatments. Mitochondrial abundance also varied between the different treatment groups (Fig. 2b). All the treatment groups showed lower mitochondrial abundance than the control group, and the bupivacaine group exhibited the lowest mitochondrial abundance, amounting to half of that of the control group. The lipid emulsion group, the puerarin group, EA-ST36, and EA-PC6 group exhibited gradually increasing mitochondrial abundances, and the ATP synthesis abilities were enhanced along with these changes in mitochondrial abundance, suggesting that their mitochondrial function was gradually recovered (Fig. 2c).

### Functional changes in mitochondria

**Fig. 3:**
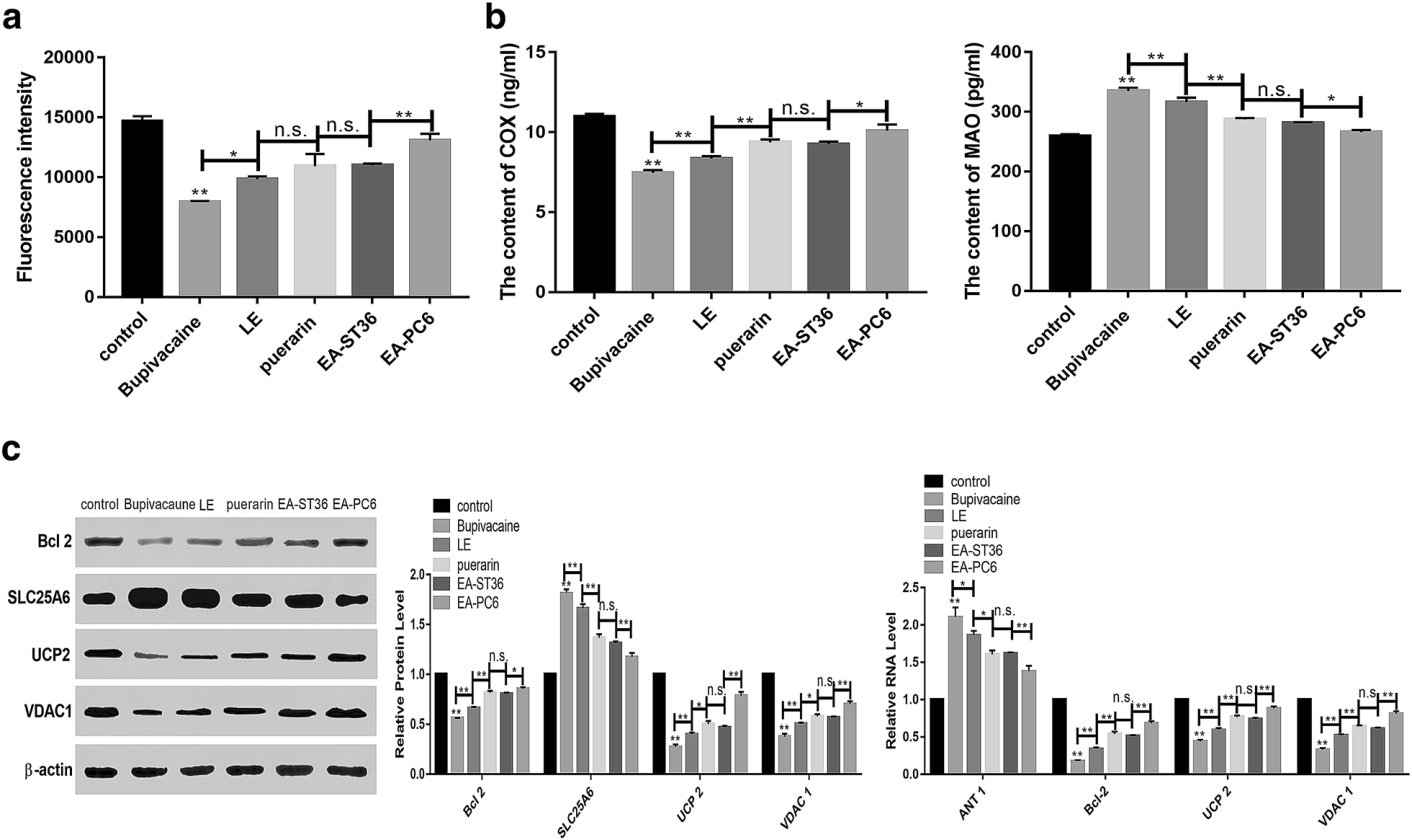
Effects of EA pre-treatment PC6 on mitochondrial functions. **a** Bupivacaine declined mitochondrial membrane potential. **b** The content of COX and MAO. **c** The expression of Bcl2, UCP2, VDAC1, SLC25A6 and ANT1

As shown in Fig. 3a, bupivacaine upregulated monoamine oxidases (MAO) activity, downregulated COX activity, and reduced MMP. This finding indicated that bupivacaine induces mitochondrial autophagy and dysfunction (Fig. 3a, b). Therapy involving lipid emulsion, puerarin, EA-ST36 and EA-PC6 pre-treatment promoted the expression of Bcl2, UCP2, and VDAC1 and decreased the expression of ANT1 and SLC25A6. After these treatments, mitochondrial dysfunction was relieved and apoptosis was reduced (Fig. 3c).

### Bupivacaine induced the oxidative stress response

**Fig. 4:**
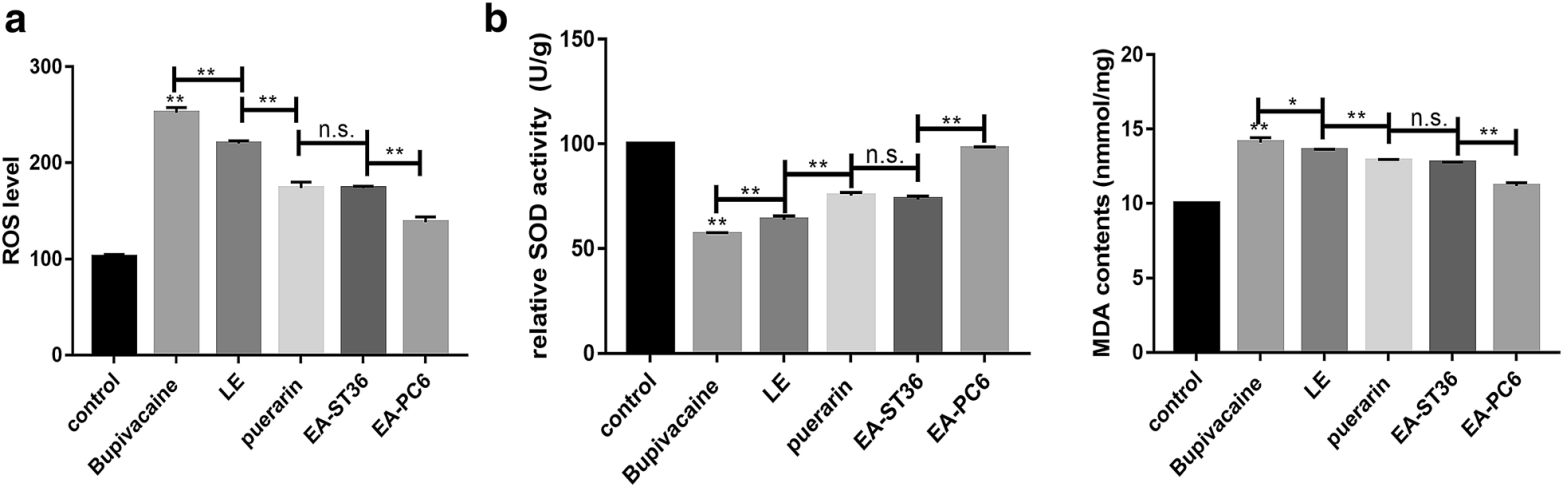
Changes of oxidase stress response of mitochondria. **a** The level of ROS. **b** The activity of SOD and level of MDA

Dysfunction of the mitochondria is closely related to ROS levels. The present results indicated that ROS production was induced by bupivacaine. All three methods of treatment increased SOD activity and decreased MDA content, playing a key role in mitochondrial protection (Fig. 4).

### Effects of bupivacaine on myocardial mitochondrial biogenesis

**Fig. 5:**
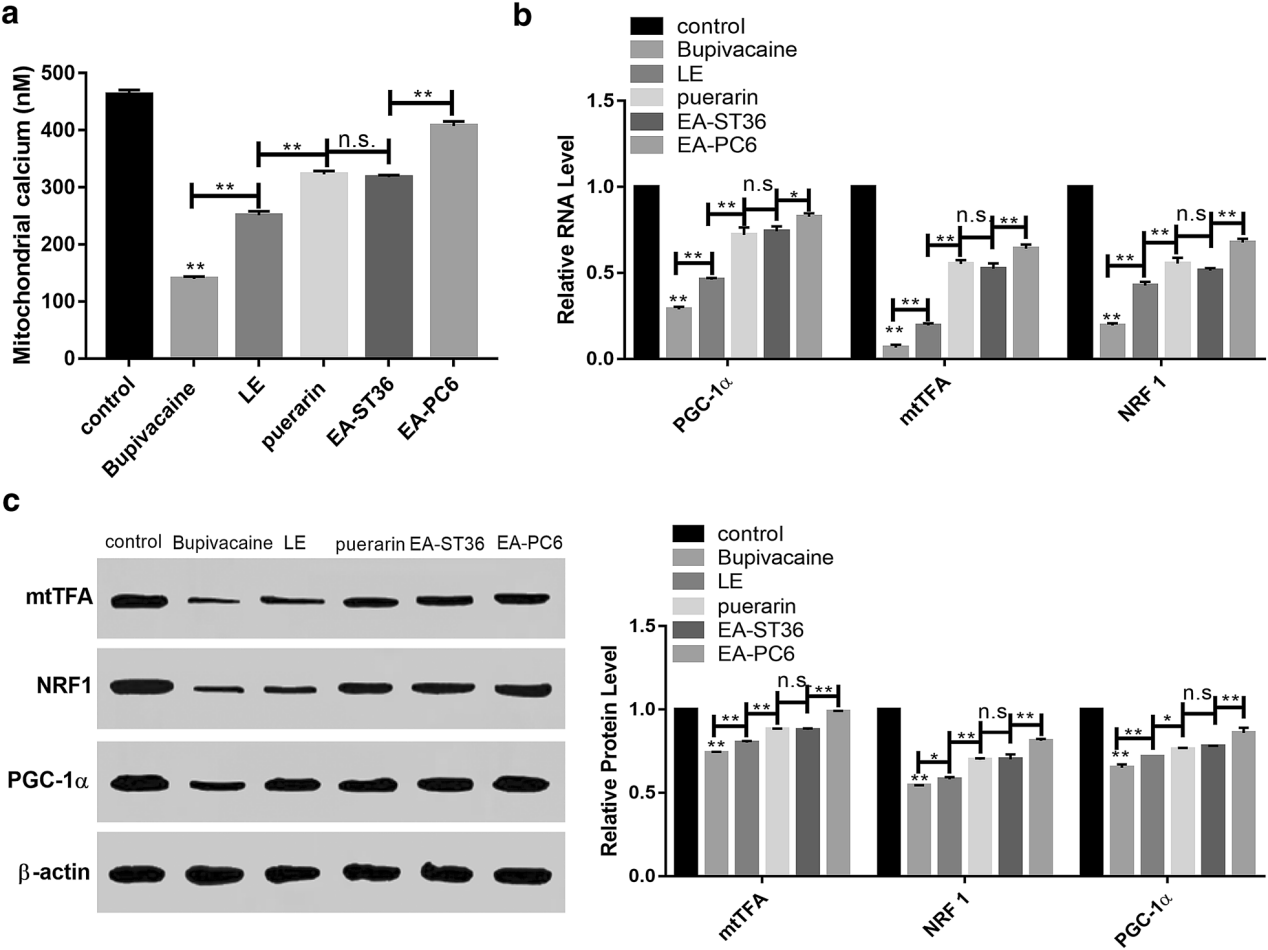
Effect of different treatment on myocardial mitochondrial biogenesis. **a** EA pre-treatment of PC6 increased the concentration of Ca2+. **b** mRNA expression of PGC, mTFA, NRF. **c** Western blot for PGC, mTFA, NRF

Mitochondrial calcium uptake significantly improved depleted cells and regulated cytosolic calcium signal [27, 28]. Our results indicated that increased Ca^2+^ levels of mitochondrial significantly upregulate the expression of PGC-1, NRF-1, and mtTFA, is one of the signals mediating mitochondrial biogenesis. The concentrations of Ca^2+^ were lowest in bupivacaine group. However, the lipid emulsion, puerarin, EA-ST36 and EA-PC6 pre-treatment groups exhibited significantly higher expression of PGC-1, mtTFA, and NRF than the bupivacaine-induced group (Fig. 5).

## Discussion

Numerous studies have confirmed that acupuncture can prevent myocardial injury. Patients who have undergone cardiac surgery have shown significantly improved heart rates, blood pressure, and rates of recovery after receiving EA stimulation [29]. Recently, researchers found that EA pre-treatment improved the survival rate of rats with myocardial ischemia–reperfusion injury with reduced apoptosis and expressions of Cyt c and cleaved caspase 3 [30, 31]. EA regulated the activation of NLRP3, polarization of macrophages, declined arrhythmia scores, improving cardiac function [18, 31]. EA played a role of anti-arrhythmia through reducing ventricular tachycardia and ventricular, and related to concentration of calcium in the cytoplasm [32, 33]. EA pre-treatment inhibits apoptosis via the mitochondria-dependent pathway [34]. In addition, EA can effectively promote angiogenesis and protect myocardial tissue from damage [31]. Previous researches have shown that the effect of electroacupuncture treatment on different acupoints was significantly different. The analgesic effect of acupuncture PC6 was better than ST36 [35, 36]. Acupuncture point of CV12 was better than PC6 and ST36 in the anorexia treatment [37]. And there was no difference in recovery of gastrointestinal function between acupuncture ST36 and the combination of acupuncture ST36 + ST37 + PC6 [38]. In present research, the results showed EA pre-treatment of PC6 to prevent bupivacaine-induced myocardial injury was the best treatment due to its highest lethal dose of bupivacaine and the superior protection effect on myocardial mitochondria.

The mitochondria, which comprise a central source of metabolism and energy production, play an important role in cellular energy metabolism. Mitochondrial injury may lead to cardiotoxicity [39]. The uncoupling of mitochondrial oxidative phosphorylation and/or inhibition of the electron transport chain leads to metabolic dysregulation in mitochondria. The cumulative release of ROS, decline in ATP synthesis, and leakage of mitochondrial Ca^2+^ may result in an inflammatory response, mitochondrial injury, which may aggravate apoptosis and lead to the death of cells surrounding the injured areas. Mitochondria-targeting treatment, considered to be an appealing strategy for the control and management of mitochondrial injury [40], has become the focus of many important research studies [41]. Previously published data have suggested that the injection of isolated viable respiration-competent myocardial mitochondria into ischemic regions prior to reperfusion may reverse post-ischemic functional deterioration and apoptosis, thereby limiting the infarct area [42].

The present findings indicated that Bcl-2 expression increased and ROS production was significantly reduced following EA pre-treatment of PC6. Similarly, calcium ions concentration of mitochondria was downregulated by bupivacaine. This is consistent with previous research [7]. We proposed that the leakage of mitochondrial calcium ions aggravated the overloaded of cytoplasmic calcium ions in arrhythmias. While, EA promoted uptake of mitochondrial calcium ions and reduced calcium overload in the cytoplasm. Moreover, the increased concentration of mitochondrial calcium promoted mitochondria biogenesis, achieving the therapeutic effect.

## Conclusions

EA pre-treatment of PC6 alleviated the mitochondrial damage caused by myocardial toxicity, enhanced the MMP, and altered concentration of Ca^2+^. All of these effects may contribute to the prevention of bupivacaine-induced cardiac arrhythmia and the improvement of myocardial metabolism.

## Data Availability

The data are available from the corresponding author on reasonable request.

## Abbreviations

ATP: Adenosine triphosphate
UCP2: Uncoupling protein 2
VDAC1: Voltage-dependent anion channel 1
SOD: Superoxide dismutases
COX: Cytochrome C oxidase
MDA: Malondialdehyde
mtTFA: Mitochondrial transcription factor A
NRF 1: Nuclear respiratory factor 1
PGC-1ɑ: Proliferator-activated receptor-gamma coactivator -1
SLC25A6: ADP/ATP translocase 3, solute carrier family 25, member 6
MRCC-I: Mitochondrial respiratory chain complex I
MIM: Mitochondrial inner membranes
MMP: Mitochondrial membrane potential
ROS: Reactive oxygen species
ERK: Extracellular signal-regulated kinase
PVC: Premature ventricular contractions
VT: Ventricular tachycardia
ELISA: Enzyme linked immunosorbent assay
MAO: Monoamine oxidases
ANT 1: Adenine nucleotide translocator 1
